# 低剂量化疗联合酪氨酸激酶抑制剂诱导治疗Ph染色体阳性急性淋巴细胞白血病的疗效和安全性

**DOI:** 10.3760/cma.j.issn.0253-2727.2022.07.004

**Published:** 2022-07

**Authors:** 欢 王, 婷 赵, 利娟 胡, 宗儒 李, 浩 江, 亚溱 秦, 悦云 赖, 红霞 石, 晓军 黄, 倩 江

**Affiliations:** 北京大学人民医院、北京大学血液病研究所、国家血液系统疾病临床医学研究中心，北京 100044 Beijing University People's Hospital, Peking University Institute of Hematology, National Clinical Research Center for Hematologic Disease, Beijing 100044, China

**Keywords:** 白血病，淋巴细胞，急性，Ph阳性, 诱导治疗, 酪氨酸激酶抑制剂, Leukemia, lymphoblastic, acute, Philadelphia chromosome positive, Induction therapy, Tyrosine kinase inhibitors

## Abstract

**目的:**

探讨低剂量化疗联合酪氨酸激酶抑制剂（TKI）作为Ph染色体阳性急性淋巴细胞白血病（Ph^+^ ALL）诱导治疗方案的有效性与安全性。

**方法:**

回顾性分析2008年1月1日至2021年7月31日北京大学人民医院收治的217例初诊Ph^+^ ALL患者的临床资料，通过logistics回归和Cox回归分析，比较低剂量化疗与常规剂量化疗的疗效及不良反应。

**结果:**

研究纳入217例患者，中位年龄38（10～69）岁，低剂量化疗组78例，常规剂量化疗组139例。低剂量化疗组与常规剂量化疗组相比，4周完全缓解（CR）率（98.7％对97.0％，*P*＝0.766）及总CR率（100％对100％，*P*＝1.000）差异均无统计学意义。多因素分析显示，化疗剂量对无病生存率、总生存率无显著影响。而两组诱导化疗期间感染发生率（*OR*＝0.444，95％ *CI* 0.227～0.866，*P*＝0.017）、粒细胞缺乏持续时间（*OR*＝0.272，95％ *CI* 0.128～0.576，*P*＝0.001）、PLT<20×10^9^/L持续时间（*OR*＝0.487，95％ *CI* 0.232～1.022，*P*＝0.057）及红细胞悬液输注量（*OR*＝0.309，95％ *CI* 0.147～0.651，*P*＝0.002）差异有统计学意义，低剂量组均显著低或短于常规剂量化疗组。

**结论:**

对于Ph^+^ ALL患者，低剂量化疗联合TKI作为一线诱导治疗的疗效与常规剂量化疗相当，且安全性好。

## Abstract (en)

**Objective:**

The study aims to explore the efficacy and safety of low-dose chemotherapy combined with tyrosine kinase inhibitor (TKI) as an induction therapy for Philadelphia-chromosomal-positive acute lymphoblastic leukemia (Ph^+^ ALL).

**Methods:**

The data of the consecutive newly diagnosed patients with Ph^+^ ALL were reviewed. The efficacy and safety of low-dose chemotherapy and conventional-dose chemotherapy combined with TKI were compared.

**Results:**

A total of 217 patients with a median age of 38（10–69）years old were included in this study. 78 patients were in the low-dose chemotherapy group, and 139 patients were in the conventional-dose chemotherapy group. There were no significant differences in the 4-week complete remission（CR）rate（98.7％ *vs* 97.0％, *P*＝0.766）and overall CR rate（100％ *vs* 100％, *P*＝1.000）between the two groups. Multivariate analyses showed that the chemotherapy intensity was not related to the disease-free survival rate and overall survival rate. However, the lower incidence of infection（*P*＝0.017）, the shorter duration of neutropenia（*P*＝0.001）and PLT<20×10^9^/L（*P*＝0.057）, and the lower red blood cell transfusion volume（*P*＝0.002）were more common in the low-dose chemotherapy group than in the conventional-dose chemotherapy group.

**Conclusion:**

The low-dose chemotherapy is superior to the conventional-dose chemotherapy combined with TKI as induction therapy in Ph^+^ ALL with similar efficacy but is safer.

酪氨酸激酶抑制剂（TKI）联合化疗使Ph染色体阳性急性淋巴细胞白血病（Ph^+^ ALL）患者的预后显著改善，5年总生存（OS）率升至40％～70％[1]–[2]，是目前Ph^+^ ALL的标准治疗方法[3]–[4]。后续联合移植可进一步改善患者的长期生存[5]–[6]。国外已有研究报道，与标准剂量化疗相比，Ph^+^ ALL患者采用低剂量化疗方案联合TKI，完全缓解（CR）率未明显降低，但早期死亡率明显下降[7]。国内相关研究较少，仅有少数小样本病例报道[8]。为此，本研究中我们回顾性分析本中心数据，对比分析TKI联合低剂量化疗与常规剂量化疗方案的有效性及安全性。

## 病例与方法

一、病例

收集2008年1月至2021年7月北京大学人民医院血液科连续收治的初诊Ph^+^ALL患者217例。所有患者均符合细胞形态学、免疫学、细胞遗传学及分子生物学（MICM）诊断标准。染色体核型分析或荧光原位杂交（FISH）检测到t（9；22）和（或）实时荧光定量逆转录聚合酶链反应（RQ-PCR）检测到BCR-ABL融合基因诊断为Ph^+^ALL。

二、治疗

1. 诱导化疗：常规剂量化疗方案为VDCP［长春地辛（VDS）4 mg，第1、8、15、22天；柔红霉素（DNR）45 mg/m^2^，第 1～3天；环磷酰胺（CTX）750 mg/m^2^，第1天；泼尼松（Pred）1 mg·kg^−1^·d^−1^× 21 d，第21天开始逐渐减量，1周减停］。低剂量化疗方案为VP（VDS 4 mg，第1、8、15、22天；Pred 1 mg·kg^−1^·d^−1^×21 d，第21天开始逐渐减量，1周减停）。初始TKI包括伊马替尼400 mg/d或达沙替尼100 mg/d，确诊后即开始TKI治疗，持续应用于整个化疗全程。

2. 巩固化疗：巩固化疗方案为改良Hyper-CVAD方案[9]+TKI。第1、3、5、7疗程采用B方案，第2、4、6、8疗程采用A方案。B方案：甲氨蝶呤 1 g/m^2^，第1天；阿糖胞苷1 g/m^2^（对于>60岁的患者减量为0.5 g/m^2^），每12 h 1次，第2～3天。A方案：CTX每次300 mg/m^2^，每12 h 1次，第1～3天；地塞米松40 mg/d，第1～4、11～l4天；VDS 4 mg，第4、11天；表阿霉素60 mg/m^2^，第4天。

3. 移植：有移植条件的患者在巩固治疗第2疗程结束后接受移植，供者来源包括同胞全相合供者、非血缘全相合供者和单倍型供者。无移植条件患者在接受至少8个疗程巩固治疗后进入维持治疗。具体移植方案参见文献[10]。

4. 维持治疗：巩固治疗结束后开始维持治疗，方案为TKI+VP，每月1个疗程，直至缓解后2～2.5年。

5. 中枢神经系统白血病（CNSL）预防和（或）治疗：鞘内注射阿糖胞苷50 mg+地塞米松5 mg。拟行移植的患者完成6次，持续化疗的患者完成16次。确诊CNSL的患者，在阿糖胞苷+地塞米松基础上加甲氨蝶呤10 mg，每周2次，直至脑脊液正常，以后每周1次×4～6周。

6. 支持治疗：骨髓抑制期若出现中性粒细胞缺乏，给予G-CSF皮下注射和预防性抗感染（细菌+真菌）治疗。若HGB<70 g/L或PLT<20×10^9^/L时予输注悬浮红细胞或单采血小板。

三、监测

诱导治疗开始后定期监测血常规和血生化常规。诱导治疗第28天及巩固治疗每疗程结束后监测骨髓细胞形态及BCR-ABL mRNA水平。BCR-ABL mRNA检测采用常规RQ-PCR方法，以ABL作为内参基因，BCR-ABL mRNA水平以BCR-ABL/ABL基因转录本拷贝数×100％表示[11]。BCR-ABL检测阴性定义为BCR-ABL mRNA拷贝数为0。若治疗过程中出现疾病复发或BCR-ABL融合基因水平持续增高或由低转高时，以Sanger测序技术进行ABL激酶突变的分析。

四、疗效判定标准及随访

CR定义为：①外周血无原始细胞，无髓外白血病[12]；②骨髓三系造血恢复，原始细胞<5％；③中性粒细胞绝对计数（ANC）>1.0×10^9^/L；④PLT>100×10^9^/L；⑤4周内无复发。复发定义为已取得CR的患者外周血或骨髓又出现原始细胞（比例>5％），或出现髓外白血病。以217例患者发病时骨髓BCR-ABL mRNA水平为基线，分析CR时BCR-ABL mRNA下降的对数级。主要分子学反应（MMR）定义为BCR-ABL mRNA较治疗前下降至少3个对数级。完全分子学反应（CMR）定义为BCR-ABL检测阴性。无病生存（DFS）期：获得CR患者，从CR之日至复发或死亡或末次随访之日。总生存（OS）时间：所有患者从诊断之日至死亡或末次随访之日。通过门诊复诊或电话问诊等方式进行随访。随访截止日期为2021年8月31日。

五、统计学处理

对于分类变量使用卡方检验，对于连续变量使用Kruskal-Wallis检验。使用logistics回归分析二分类变量结局（CR时BCR-ABL mRNA下降≥2个对数级、感染发生率、粒细胞缺乏持续时间、PLT<20×10^9^/L持续时间、红细胞输注量、血小板输注量）的影响因素，使用Cox回归分析DFS和OS的影响因素。在logistics回归和Cox回归的单因素分析中，以中位数作为界值将连续变量转化为二分类变量，纳入分析的自变量包括性别、年龄、WBC、HGB、PLT、肝脾淋巴结肿大、染色体核型、骨髓原始细胞、外周血原始细胞、IKZF1突变、BCR-ABL转录本类型、初始TKI、诱导方案、移植、CR时BCR-ABL mRNA下降的对数级、第1疗程巩固治疗后BCR-ABL mRNA下降的对数级、第2疗程巩固治疗后BCR-ABL mRNA下降的对数级。将单因素分析中*P*<0.2的变量纳入多因素分析。使用Log-rank分析比较不同分组之间DFS和OS的差异，用Kaplan-Meier曲线呈现。以*P*<0.05为差异有统计学意义。采用SPSS 21.0统计学软件进行统计分析。

## 结果

一、患者特征

217例初诊的Ph^+^ ALL患者根据诱导治疗方案分为两组，其中低剂量化疗组78例，常规剂量化疗组139例。低剂量化疗组联合伊马替尼22例，联合达沙替尼56例。常规剂量化疗组联合伊马替尼107例，联合达沙替尼29例。与常规剂量组相比，低剂量化疗组患者年龄更高（*P*＝0.035）、有附加染色体核型的比例更高（*P*＝0.027）、联合达沙替尼的患者更多（*P*<0.001），其他临床特征见表1。

**表1:** 217例初诊Ph染色体阳性急性淋巴细胞白血病患者临床特征

特征	患者总体（217例）	低剂量化疗组（78例）	常规剂量化疗组（139例）	*χ*^2^/*z*值	*P*值
男性［例（％）］	128（59.0）	47（60.3）	81（58.7）	0.047	0.776
年龄［岁，*M*（范围）］	38（10～69）	40（16～69）	37（10～68）	−2.114	0.035
WBC［×10^9^/L，*M*（范围）］	26.2（0.5～487.6）	24.1（1.4～348.8）	27.2（0.5～487.6）	−0.462	0.671
HGB［g/L，*M*（范围）］	98.0（44.0～198.0）	105.0（44.0～198.0）	93.0（46.0～153.0）	−1.945	0.050
PLT［×10^9^/L，*M*（范围）］	40.0（0.2～320.0）	43.0（5.0～304.0）	39.0（0.2～320.0）	−0.022	0.914
肝脾淋巴结肿大［例（％）］	33（15.2）	64（82.1）	120（86.3）	0.653	0.400
染色体［例（％）］				2.032	0.006
正常核型	35（16.4）	11（14.3）	24（17.5）		
标准Ph核型	63（29.4）	16（20.8）	47（34.3）		
有附加核型	101（47.2）	48（62.3）	53（38.7）		
未见分裂象	15（7.0）	2（2.6）	13（9.5）		
骨髓原始细胞［％，*M*（范围）］	88（17～98）	89（17～98）	88（21～98）	−0.686	0.493
外周血原始细胞［％，*M*（范围）］	52（0～98）	50（0～98）	53（0～98）	−0.378	0.694
IKZF1缺失阳性［例（％）］	99（58.9）	42（56.8）	57（60.6）	0.388	0.612
初始BCR-ABL［％，*M*（范围）］	103.3（9.9～430.8）	103.3（24.8～430.8）	102.7（9.9～203.5）	−1.187	0.183
BCR-ABL转录本类型［例（％）］				0.030	0.893
P190型	149（68.7）	54（69.2）	95（68.3）		
P210型	68（31.3）	24（30.8）	44（31.7）		
初始TKI［例（％）］				60.206	<0.001
伊马替尼	129（60.3）	22（28.2）	107（78.7）		
达沙替尼	85（39.7）	56（71.8）	29（21.3）		

注：低剂量化疗组：采用VP（长春地辛、泼尼松）方案；常规剂量化疗组：采用CODP（长春地辛、柔红霉素、环磷酰胺、泼尼松）方案；TKI：酪氨酸激酶抑制剂

二、治疗反应

1. 血液学缓解率：217例患者中，早期死亡5例（2.3％），207例（97.6％）在4周诱导化疗后获得CR，1例在1个疗程未缓解再次诱导后失访，最终可评估的211例患者均获得CR。低剂量化疗组与常规剂量化疗组4周CR率（98.7％对97.0％，*P*＝0.766）及总CR率（100％对100％，*P*＝1.000）差异均无统计学意义。

2. 分子学反应：4周诱导化疗后，CR时BCR-ABL mRNA下降的对数级中位值［2.7（0～4.2）对1.8（0.1～4.1），*P*＝0.003］、CR时BCR-ABL mRNA下降≥2个对数级患者比例（43.4％对23.5％，*P*＝0.003）及CR时获得MMR患者比例（65.8％对42.5％，*P*＝0.001），低剂量化疗组显著高于常规剂量化疗组。巩固1个疗程［3.4（0.1～4.4）对3.0（0.2～4.2），*P*＝0.061］及巩固2个疗程［3.7（0～4.5）对3.4（0～4.6），*P*＝0.081］后BCR-ABL mRNA下降程度，低剂量化疗组有高于常规剂量化疗组的趋势。两组CR时获得CMR患者比例差异无统计学意义（7.9％对7.6％，*P*＝0.946）。

分析患者诊断时特征和不同诱导化疗方案与分子学反应的关系。多因素分析显示，男性、标准染色体核型、骨髓原始细胞≥88％、外周血原始细胞<52％、低剂量诱导化疗与CR时BCR-ABL mRNA下降≥2个对数级有显著相关性（表2）。分别在接受伊马替尼和达沙替尼的患者中进行分析，多因素分析显示，接受伊马替尼的患者，治疗前WBC<27×10^9^/L、骨髓原始细胞≥88％ 与CR时BCR-ABL mRNA下降≥2个对数级有显著相关性。接受达沙替尼的患者，男性、外周血原始细胞<52％与CR时BCR-ABL mRNA下降≥2个对数级有显著相关性。在这两组患者中，化疗剂量均与CR时BCR-ABL mRNA下降≥2个对数级无显著相关性（表3、表4）。

**表2:** 影响Ph染色体阳性急性淋巴细胞白血病患者CR时BCR-ABL mRNA下降、DFS率与OS率的多因素分析结果

因素	CR时BCR-ABL mRNA下降≥2个对数级		DFS		OS	
	*OR*（95％ *CI*）	*P*值	*HR*（95％ *CI*）	*P*值	*HR*（95％ *CI*）	*P*值
女性（是，否）	0.50（0.26～0.96）	0.038	1.91（1.13～3.23）	0.016		
WBC≥27×10^9^/L（是，否）	0.50（0.25～1.02）	0.058				
HGB<88 g/L（是，否）						
PLT<44×10^9^/L（是，否）					2.51（1.46～4.32）	0.001
染色体核型		0.031				
正常核型						
未见分裂象	0.06（0.01～0.54）	0.013				
标准Ph核型	1.12（0.41～3.05）	0.823				
有附加核型	0.62（0.24～1.59）	0.318				
骨髓原始细胞≥88％（是，否）	2.69（1.32～5.49）	0.006				
外周血原始细胞≥52％（是，否）	0.44（0.20～0.96）	0.039	2.48（1.49～4.12）	<0.001	1.97（1.17～3.32）	0.011
IKZF1缺失阳性（是，否）						
P190型（P190型，P210型）					1.95（1.08～3.52）	0.026
常规剂量化疗（常规剂量，低剂量）	0.38（0.19～0.74）	0.004				
移植（是，否）			0.12（0.07～0.20）	<0.001	0.18（0.10～0.31）	<0.001

注：CR：完全缓解；DFS：无病生存；OS：总生存；低剂量化疗：VP（长春地辛、泼尼松）方案；常规剂量化疗：CODP（长春地辛、柔红霉素、环磷酰胺、泼尼松）方案

**表3:** 影响伊马替尼组患者CR时BCR-ABL mRNA下降、DFS率与OS率的多因素分析结果

因素	CR时BCR-ABL mRNA下降≥2个对数级		DFS		OS	
	*OR*（95％ *CI*）	*P*值	*HR*（95％ *CI*）	*P*值	*HR*（95％ *CI*）	*P*值
女性（是，否）			2.98（1.56～5.68）	0.001		
WBC≥27×10^9^/L（是，否）	0.37（0.16～0.85）	0.018			2.16（1.14～4.07）	0.018
HGB<88 g/L（是，否）						
PLT<44×10^9^/L（是，否）					2.41（1.29～4.52）	0.006
骨髓原始细胞≥88％（是，否）	2.96（1.30～6.74）	0.010				
外周血原始细胞≥52％（是，否）			2.45（1.34～4.46）	0.003		
IKZF1缺失阳性（是，否）						
P190型（P190型，P210型）					2.29（1.12～4.69）	0.024
常规剂量化疗（常规剂量，低剂量）						
移植（是，否）			0.08（0.04～0.18）	<0.001	0.15（0.08～0.28）	<0.001

注：CR：完全缓解；DFS：无病生存；OS：总生存；低剂量化疗：VP（长春地辛、泼尼松）方案；常规剂量化疗：CODP（长春地辛、柔红霉素、环磷酰胺、泼尼松）方案

**表4:** 影响达沙替尼组患者CR时BCR-ABL mRNA下降、DFS率与OS率的多因素分析结果

因素	CR时BCR-ABL mRNA下降≥2个对数级		DFS		OS	
	*OR*（95％ *CI*）	*P*值	*HR*（95％ *CI*）	*P*值	*HR*（95％ *CI*）	*P*值
女性（是，否）	0.23（0.07～0.74）	0.014				
WBC≥27×10^9^/L（是，否）	0.36（0.12～1.04）	0.059				
HGB<88 g/L（是，否）						
PLT<44×10^9^/L（是，否）					6.59（1.89～22.95）	0.003
骨髓原始细胞≥88％（是，否）						
外周血原始细胞≥52％（是，否）	0.26（0.08～0.79）	0.018	2.95（1.08～8.09）	0.036		
IKZF1缺失阳性（是，否）						
常规剂量化疗（常规剂量，低剂量）					2.77（1.07～7.18）	0.036
移植（是，否）			0.23（0.081～0.64）	0.005		

注：CR：完全缓解；DFS：无病生存；OS：总生存；低剂量化疗：VP（长春地辛、泼尼松）方案；常规剂量化疗：CODP（长春地辛、柔红霉素、环磷酰胺、泼尼松）方案

三、诱导化疗中骨髓抑制期的不良事件和输血

1. 早期死亡：早期死亡的5例患者中，低剂量化疗组1例，常规剂量化疗组4例。3例死于重度感染，各有1例分别死于脑出血和脑梗死。

2. 骨髓抑制时间：低剂量化疗组ANC<0.5×10^9^/L及PLT<20×10^9^/L的中位持续时间显著短于常规剂量化疗组［4（0～26）d对9（0～26）d，*P*<0.001；3（0～23）d对5（0～39）d，*P*＝0.021］。根据中位数得到的界值将连续变量转化为分类变量，ANC<0.5×10^9^/L的持续时间以8 d为界值，PLT<20×10^9^/L的持续时间以4 d为界值。多因素分析显示，低剂量化疗与粒细胞缺乏低于8 d和PLT<20×10^9^/L低于4 d有显著相关性。此外，PLT≥44×10^9^/L与粒细胞缺乏低于8 d和PLT<20×10^9^/L低于4 d均有显著相关性，骨髓原始细胞<88％与粒细胞缺乏低于8 d有显著相关性，外周血原始细胞<52％与PLT<20×10^9^/L低于4 d有显著相关性（表5）。

**表5:** 影响Ph染色体阳性急性淋巴细胞白血病患者骨髓抑制时间、血制品输注量的多因素分析结果

因素	粒细胞缺乏低于8 d		PLT<20×10^9^/L低于4 d		红细胞输注量<6 U		血小板输注量<2 U	
	*OR*（95％ *CI*）	*P*值	*OR*（95％ *CI*）	*P*值	*OR*（95％ *CI*）	*P*值	*OR*（95％ *CI*）	*P*值
女性（是，否）								
WBC<27×10^9^/L（是，否）							2.63（1.35～5.11）	0.004
HGB≥88 g/L（是，否）					6.40（2.93～14.00）	<0.001		
PLT≥44×10^9^/L（是，否）	2.37（1.28～4.40）	0.006	6.68（3.20～13.92）	<0.001			5.90（3.06～11.41）	<0.001
染色体核型								
正常核型								
未见分裂象								
标准Ph核型								
有附加核型								
骨髓原始细胞<88％（是，否）	2.18（1.16～4.10）	0.015						
外周血原始细胞<52％（是，否）			2.52（1.22～5.20）	0.012	2.71（1.29～5.69）	0.008		
IKZF1阳性（是，否）								
P210型（P210型，P190型）								
低剂量化疗（低剂量，常规剂量）	5.02（2.60～9.73）	<0.001	2.42（1.73～4.98）	0.017	3.99（1.86～8.58）	<0.001		

注：低剂量化疗：VP（长春地辛、泼尼松）方案；常规剂量化疗：CODP（长春地辛、柔红霉素、环磷酰胺、泼尼松）方案

3. 感染：低剂量化疗组感染发生率为57.7％，显著低于常规剂量化疗组的71.2％（*P*＝0.047）。多因素分析显示，低剂量化疗（*OR*＝0.443，95％ *CI* 0.227～0.866，*P*＝0.017）可显著降低感染发生率。

4. 输注血制品：低剂量化疗组中位红细胞输注量显著低于常规剂量化疗组［4（0～14）U对6（0～22）U，*P*＝0.001］，而两组血小板输注量差异无统计学意义（*P*＝0.131）。根据中位数得到的界值将连续变量转化为分类变量，红细胞输注量以6 U为界值，血小板输注量以2 U为界值。多因素分析显示，低剂量化疗与红细胞输注量<6 U有显著相关性。此外，HGB≥88 g/L、外周血原始细胞<52％与红细胞输注量<6 U有显著相关性，WBC<27×10^9^/L、PLT≥44×10^9^/L与血小板输注量<2 U有显著相关性，诱导化疗方案与血小板输注量没有显著相关性（表5）。

四、复发和生存

211例获得CR患者中位随访32.2（1.2～165.6）个月，存活患者中位随访42.1（1.2～165.6）个月。151例（71.6％）接受移植，60例（28.4％）持续化疗联合TKI。低剂量化疗组更多患者接受持续化疗联合TKI（44.9％对22.3％，*P*<0.001）。211例CR患者中，69例复发，中位复发时间20.6（0.9～90.1）个月，在检测突变的54例复发患者中，42例（77.8％）检测到ABL激酶突变，其中29例（69.0％）为T315I突变。死亡63例，其中10例死于缓解期重症感染，34例死于复发，19例死于移植相关并发症。5年DFS率为53.2％，5年OS率为61.7％。低剂量化疗组5年DFS率（44.7％对56.1％，*P*＝0.142）及OS率（53.5％对61.3％，*P*＝0.661）与常规剂量化疗组比较差异无统计学意义（图1）。

**图1:**
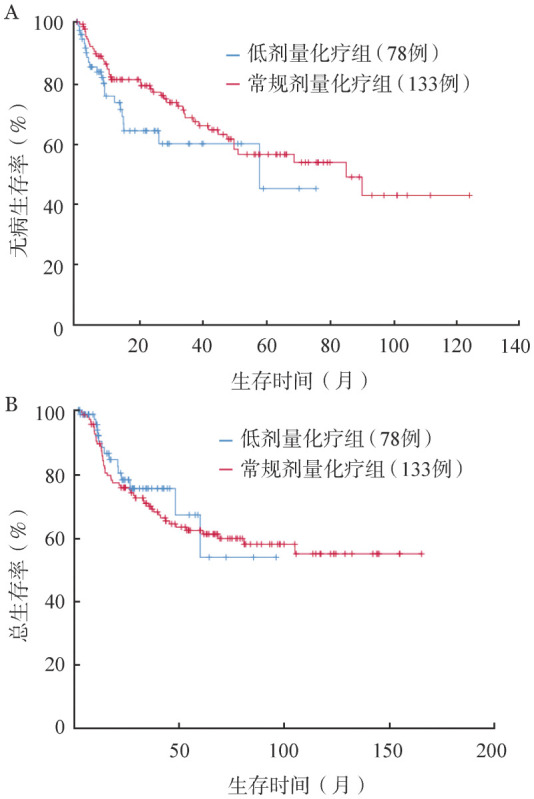
211例完全缓解患者低剂量化疗与常规剂量化疗的无病生存期（A）及总生存期（B）比较

分析患者基线特征和首次诱导化疗方案与DFS及OS的相关性。多因素分析显示，移植与更长的DFS与OS时间均显著相关。女性、外周血原始细胞≥52％与更短的DFS时间显著相关，PLT≤44×10^9^/L、外周血原始细胞≥52％、P190型与更短的OS时间显著相关。而首次诱导化疗方案不是影响DFS和OS的独立预后因素（表2）。将伊马替尼与达沙替尼进行分组分析，伊马替尼组，首次诱导化疗方案不是影响DFS和OS的独立预后因素，移植仍与更长的DFS与OS时间显著相关（表3）。达沙替尼组，首次诱导化疗方案不影响DFS，移植与更长的DFS时间显著相关，低剂量化疗与更长的OS时间显著相关（表4）。

## 讨论

本研究结果显示，治疗Ph^+^ ALL患者的诱导方案中，低剂量化疗联合TKI与常规剂量联合TKI相比，CR率、5年OS率和5年DFS率差异均无统计学意义，但常规剂量化疗组早期死亡率更高。关于低剂量与常规剂量化疗相比较的国外研究中，Chalandon等[7]报道的GRAAPH-2005临床试验是一项前瞻性随机对照研究，入组268例Ph^+^ ALL患者，中位年龄47岁，在诱导期分别接受伊马替尼联合小剂量化疗（长春新碱+地塞米松，VP方案）和伊马替尼联合Hyper-CVAD化疗，结果显示CR率分别为98％和91％，差异有统计学意义（*P*＝0.006），5年OS率分别为48％和43％，5年EFS率分别为42％和32％，差异均无统计学意义。而小剂量化疗组早期死亡率低于常规化疗组（1.7％对6.7％，*P*＝0.01）。目前国内关于这一方面的研究较少[8]，且样本量小。此外，国内及国外研究中联合方案的TKI均为伊马替尼。本研究样本量较大，年轻患者多，联合方案中TKI包括一代伊马替尼及二代达沙替尼。此外，本研究不仅分析了伊马替尼联合低剂量化疗方案的有效性，还分析了达沙替尼联合低剂量化疗方案的有效性及安全性，并进一步经多因素分析显示CR率及生存结局均与Chalandon等报道相符。本研究中，接受伊马替尼的患者多联合常规剂量化疗，接受达沙替尼的患者多联合低剂量化疗，尽管在总体人群中发现低剂量化疗与更深的分子学反应相关，但在TKI分组分析中，联合的化疗剂量与分子学反应无明显相关性。

诱导治疗相关的骨髓抑制是导致感染的重要原因，国内报道感染率可达40％～80％[13]–[14]。在GRAAPH-2005研究中，与常规剂量组相比，低剂量化疗组ANC<0.5×10^9^/L与PLT<20×10^9^/L的中位持续时间（5.5 d对13.5 d，*P*<0.001；0 d对2.5 d，*P*<0.001）及3～4级感染事件发生率（37％对58％，*P*<0.001）显著降低。Rousselot等[15]报道71例患者在诱导期接受达沙替尼联合VP方案治疗，中位年龄69岁，ANC<0.5×10^9^/L的中位持续时间为8.9 d，PLT<20×10^9^/L的中位持续时间为3 d。Foà等[16]报道53例患者在诱导期接受达沙替尼联合糖皮质激素的治疗，中位年龄53.6岁，感染率只有3.8％。以上几项研究低剂量化疗感染发生率均低于常规剂量化疗，除一项随机对照研究外，其余均为单臂研究，联合TKI为伊马替尼或达沙替尼，统计结果均为单因素分析。本研究结果显示低剂量化疗方案可显著降低感染发生率、缩短骨髓抑制时间，与报道一致。更为重要的是，本研究通过多因素分析证实，无论联合伊马替尼或达沙替尼，低剂量化疗方案是影响感染发生率、骨髓抑制时间的独立保护因素。此外，经多因素分析还发现，无论对老年还是年轻患者，低剂量化疗方案是影响红细胞输注量的独立保护因素，在血制品匮乏的时代，减少输血量，保证患者治疗安全尤为重要。

本研究的缺陷包括：①为回顾性研究，化疗方案联合TKI不完全统一。低剂量化疗组患者随访时间短，两组后续治疗接受移植的患者比例有偏差，可能影响结局。但是我们用多因素分析进行调整，结果仍提示低剂量化疗方案与常规剂量化疗方案不是DFS及OS的独立影响因素。②低剂量化疗组多联合达沙替尼（71.8％），常规剂量化疗组多联合伊马替尼（78.7％），两者有极强的相关性（*P*<0.001），所以我们试图用倾向性配对分析去匹配两组，因例数过少无法分析。因此我们无法分析达沙替尼与伊马替尼作为不同种类初始TKI对治疗反应和结局的影响。

总之，本研究结果显示，与常规化疗方案联合TKI相比，低剂量化疗方案联合TKI的缓解率和长期生存率相当，更重要的是安全性好，是一线Ph^+^ ALL诱导治疗方案较好的选择。
